# Measurement Performance of Two Continuous Tissue Glucose Monitoring Systems Intended for Replacement of Blood Glucose MonitoringParts of the data have previously been presented at the 77th Scientific Sessions of the American Diabetes Association in San Diego, CA; June 9–13, 2017 and at the 17th Annual Diabetes Technology Meeting in Bethesda, MD, November 2–4, 2017.Trial number: DRKS00011920; registered at the Deutsches Register Klinischer Studien (German clinical trials register), an approved Primary Register in the WHO International Clinical Trials Registry Platform.

**DOI:** 10.1089/dia.2018.0105

**Published:** 2018-08-01

**Authors:** Guido Freckmann, Manuela Link, Stefan Pleus, Antje Westhoff, Ulrike Kamecke, Cornelia Haug

**Affiliations:** Institut für Diabetes-Technologie Forschungs- und Entwicklungsgesellschaft mbH an der Universität Ulm, Ulm, Germany.

**Keywords:** Measurement accuracy, Blood glucose monitoring, Blood glucose monitoring replacement, Continuous glucose monitoring, Nonadjunctive use.

## Abstract

***Background:*** Currently, two systems for continuous tissue glucose monitoring (CGM) (Dexcom^®^ G5 [DG5] and FreeStyle Libre [FL]) are intended to replace blood glucose monitoring (BGM) and, according to manufacturer labeling, are distributed as such in some jurisdictions, including the United States and the European Union.

***Methods:*** The measurement performance of these two systems in comparison with a BGM system was analyzed in a 14-day study with 20 participants comprising study site visits, which included phases of induced rapid glucose changes, and home use phases. Performance analysis was mainly based on deviations between CGM readings and BGM results. Sensor-to-sensor precision was also analyzed.

***Results:*** Approximately 25% of DG5 and FL results showed differences from BGM results exceeding 15 mg/dL or 15% (at glucose concentration below or above 100 mg/dL, respectively) at times of therapeutic decisions, and ∼5% of differences exceeded 30 mg/dL or 30%. Performance was different depending on the setting (study site visits, home use phases, and phases of induced rapid glucose changes). In consensus error grid (CEG) analysis, both systems showed >99.5% of results within the clinically acceptable zones A and B.

***Conclusions:*** In this study, both systems showed deviations from blood glucose (BG) measurements, the current standard approach in diabetes therapy. Although a large percentage of results was found in CEG zones A and B, for approximately one in four therapeutic decisions, CGM and BG readings differed by at least 15 mg/dL or 15%. Such deviations should be taken into account when using CGM systems.

## Background

Modern diabetes therapy aims for near-normal levels of blood glucose (BG). The current standard glucose monitoring regimen for patients with diabetes mellitus is capillary blood sampling to achieve tight monitoring. Although patients may perform several BG measurements per day, glucose records are incomplete, for example, during sleep.

In contrast, a continuous tissue glucose monitoring (CGM) system can, in principle, provide a more complete picture with a larger number of glucose readings and thus support the assessment of the metabolic control, detect trends and patterns, and form the basis for individualized diabetes regimes. CGM systems typically not only show a current glucose reading but also glucose trend information or a graph of previous glucose levels (glucose curve).

The measurement performance of various commercially available CGM systems has been investigated by different study groups in recent years,^1–9^ and CGM systems are an important component in artificial pancreas systems.^10–12^

Recently, the Dexcom G5^®^ (DG5) Mobile (Dexcom, Inc., San Diego, CA), a real-time CGM (rtCGM) system, and the FreeStyle Libre (FL; Abbott Diabetes Care, Alameda, CA) system, a so-called flash glucose monitoring system, were approved by the Food and Drug Administration (FDA) for replacement of BG measurements, that is, for nonadjunctive use, with some exceptions as mentioned in the devices' labeling. Both systems are also marketed in the European Union (EU) to replace BG measurements, with slight differences in the device labeling in case of FL (e.g., 14 days of use in the EU vs. 10 days in the United States; 1-h warm-up in the EU vs. 12-h warm-up in the United States; additional icon for suggested BG test in the United States).

In this study, the performance of these two systems was investigated under daily life conditions and in a clinical setting incorporating parts of the POCT05-A guideline.^13^ Because both investigated systems are intended to replace BG measurements, the objective of the study was a head-to-head system performance evaluation of the two systems in comparison with a blood glucose monitoring system (BGMS), the current standard approach to glucose monitoring.

## Methods

This open-label, mono-center, single-arm, investigator-initiated clinical trial was performed in compliance with the Declaration of Helsinki (revised 2013, Fortaleza, Brazil), with Good Clinical Practice, and with local laws and regulations at the Institut für Diabetes-Technologie Forschungs- und Entwicklungsgesellschaft mbH an der Universität Ulm (IDT), Ulm, Germany, between March 2016 and October 2016. The responsible independent Ethics Committee approved the study protocol before any study procedures were performed and before any participants were recruited. The trial was exempted from regulatory approval by the competent authority. The trial was registered in the German Clinical Trial Register (“Deutsches Register Klinischer Studien,” [DRKS]) with the registration number DRKS00011920.

### Investigational devices

This study evaluated readings of two tissue glucose monitoring systems, the rtCGM system DG5 Mobile (Dexcom, Inc.) and the FL (Abbott Diabetes Care) system, in comparison with BGMS results. The investigational devices used in the study were Conformité-Européenne-marked and used as intended.

After insertion of glucose sensors in the subcutaneous tissue, participants got access to the current glucose level either in real-time (DG5) or by scanning with a suitable device (FL). Furthermore, the systems provided a glucose curve and displayed trend arrows to allow a forecast of the future course of glucose concentrations.

Participants were advised to not take medication containing acetaminophen or ascorbic acid or salicylic acid because of possible interference with CGM readings.

### Comparison measurement methods

A BGMS served as main comparison method because it is the current standard approach to glucose monitoring in diabetes therapy, and because both DG5 and FL are intended to replace blood glucose monitoring (BGM). In addition, the comparison method was intended to be the same during study site visits and during home use phases, for which laboratory analyzer measurements were not feasible.

To potentially minimize a possible bias caused by the manufacturer's reference method used to factory-calibrate FL, a FreeStyle Freedom Lite (Abbott Diabetes Care) BGMS was used to calibrate DG5. The BGMS was characterized beforehand in a study based on ISO 15197:2013,^14^ an internationally accepted standard stipulating requirements for BGMS intended for self-monitoring. In that characterization, the two test strip lots used in this study showed 100% and 99.5% of results within 10 mg/dL (at glucose concentrations <100 mg/dL) or 10% (at glucose concentrations ≥100 mg/dL) of the manufacturer's reference method, with only minimal bias (−1.2% and −1.5%).

Additional comparison measurements in venous blood samples were performed on a hexokinase (HK)-based laboratory glucose analyzer (Cobas Integra^®^ 400 Plus; Roche Instrument Center, Rotkreuz, Switzerland) that conforms to the traceability requirements of ISO 17511^15^ according to manufacturer's information.

### Study design

**FIG. 1.**
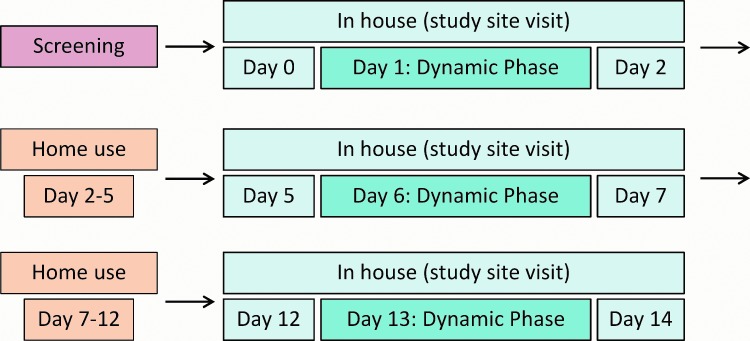
Study timeline.

After written informed consent was obtained from potential participants, they were physically examined in a screening visit. After screening, 20 participants with type 1 diabetes met all inclusion criteria and none of the exclusion criteria (see Supplementary Data available at http://online.liebertpub.com/doi/suppl/10.1089/dia.2018.0105) and were enrolled in a 14-day experiment phase. Participants visited the study site on three separate occasions (experiment days 0–2, days 5–7, and days 12–14) with home use phases in between (Fig. 1). In the EU, FL is intended for 14 days of use, as opposed to 10 days of use in the United States, so that the 14-day experiment phase covered one sensor wear time of FL.

On the first study day (experiment day 0), participants were equipped with the DG5 and FL systems. A study physician applied two sensors per system: one FL sensor on each upper arm and one DG5 sensor on the left and right side of the abdomen, respectively. DG5 sensors were routinely replaced after ∼7 days of sensor use to achieve a total sensor wear time of ∼14 days for both systems. A total of four DG5 sensors and two FL sensors were used by each participant.

During the study site visits, BG measurements were performed with the study BGMS in duplicate at least once per hour between 06:00 and 24:00 and once at night. BG duplicates had to differ no more than 10 mg/dL or 10% (below or above 100 mg/dL, respectively), or a third measurement had to be performed. For DG5 calibration and for therapeutic decisions, the last obtained BG measurement result of these duplicates or triplicates was used to facilitate study procedures during the home use phases. In parallel to each BG measurement, sensor readings and trend arrows of all four sensors were documented in a diary.

On experiment days 1, 6, and 13, rapid glucose changes with values in the hyper- and hypoglycemic range were induced by intake of a breakfast meal with high glycemic index and delaying and increasing the insulin bolus dose. From 30 min before breakfast up to 5 h after breakfast (“dynamic phase”), BG was monitored every 15 min in capillary blood samples with the study BGMS and in venous blood samples with the HK-based method.

During home use phases, participants monitored BG at least five times per day (after getting up, before each major meal, and at 23:00). BG measurements and documentation were performed identical to procedures during study site visits with the exception of not documenting trend arrows.

### Data analysis

Performance parameters, technical outcomes, and safety outcomes were defined.

For performance parameters, evaluation units were based either on aggregated data (i.e., all glucose data), on data of individual sensors (i.e., data grouped by *n* = 80 DG5 sensors, each including data from up to 7 days of sensor use, or *n* = 40 FL sensors, each including data from up to 14 days of sensor use), on application-site specific data (i.e., data grouped by left/right side of each individual participant's body, *n* = 40 (20 left abdomen or upper arms and 20 right abdomen or upper arms), each including data from up to 14 days of use), and on participant-specific data (i.e., data grouped by participant, *n* = 20) (Supplementary Fig. S1). Grouping by application site was performed so that the CGM systems could be compared based on 14 days of continuous use.

Data sets were based on complete experiments (14 days), on in-house phases (∼3 × 48 h), on home use phases (∼72 and 120 h), on dynamic phases (∼3 × 5 h), and on therapeutic decisions. A therapeutic decision was defined as a BG measurement that was performed at the time of meal intake and/or insulin delivery.

Technical and safety outcomes were based on data of the complete study. Device deficiencies (DD) and premature sensor removal were analyzed as technical outcome; adverse events (AE), and adverse device effects (ADE), that is, AE with possible, probable, or definitive causal relationship to an investigational device, were analyzed as safety outcomes.

For FL, analyses were performed for scanned data and for continuously stored data points, separately.

Performance parameter analyses were based on comparison with measurement results from the study BGMS, or on sensor-to-sensor comparisons, or on comparison with measurement results from the HK-based method. The warm-up periods of 2 h for DG5 and 1 h for FL were excluded, because the CGM systems did not provide data during that time.

This study was conducted in an exploratory manner.

The primary endpoint of this trial was the percentage of differences or relative differences between sensor readings and BG measurements exceeding specific thresholds, similar to an analysis performed by Leelarathna et al.,^16^ at times of therapeutic decisions. This data set was used to highlight the possible impact for a diabetes patient when switching from a BGMS to a CGM system. Secondary analyses included similar calculations based on other data sets, and the mean absolute difference (MAD) and mean absolute relative difference (MARD), and precision absolute difference (PAD), and precision absolute relative difference (PARD; i.e., sensor-to-sensor differences).^17^ For these analyses, differences or absolute differences were calculated at glucose concentrations <100 mg/dL, and relative differences or relative absolute differences were calculated at glucose concentrations ≥100 mg/dL. This approach was chosen because the investigated CGM systems were intended to replace measurements with BGMS, and ISO 15197:2013,^14^ an internationally accepted standard stipulating requirements for BGMS intended for self-monitoring, requires the same cutoff for differences and relative differences. MARD and PARD results for the complete glucose concentration range are provided in the Supplementary Data. A consensus error grid (CEG) analysis was performed.^18^ An additional analysis focused on the number and percentage of differences between CGM readings and BG measurement results within specific accuracy limits based on the system accuracy criteria of ISO 15197:2013 (Supplementary Table S1).^14^

For primary endpoint and similar secondary endpoint analyses, scanned FL data (FLscan) were used if the scan was performed within ±3 min of a BG measurement, and for DG5, the last stored ( = displayed) value within up to 5 min before the BG measurement was used (“last observed carried forward” [LOCF] or “step function” approach). This data selection was used to simulate which data the user would have used if she/he had not performed a BG measurement.

For MAD/MARD and PAD/PARD analyses, continuously stored FL data (FLcont) and DG5 data were linearly interpolated to one value per minute, because this is a common approach in estimating measurement performance of CGM systems. In addition, the same values as used in the primary endpoint analysis (using the LOCF approach) were used to calculate MAD/MARD. FLscan data were used as recorded if the scan was performed within ±3 min of a BG measurement.

## Results

### Participants

A total of 20 participants (8 female, 12 male) were enrolled in the trial. All participants had type 1 diabetes and were either on multiple daily injections (30%) or continuous subcutaneous insulin infusion (70%). Participants were between 21 and 64 years of age, and the average age was 39.0 ± 13.2 years (mean ± standard deviation). Their diabetes was diagnosed between 1 and 45 years before enrollment, with an average of 21.2 ± 10.8 years. Body mass index was 26.3 ± 3.9 kg/m^2^ and ranged from 20.5 to 37.5 kg/m^2^. At the screening visit, the average glycated hemoglobin (HbA1c) level was 7.2% ± 1.1% (55.6 ± 11.6 mmol/mol), ranging from 5.7% to 9.6% (38.8–81.4 mmol/mol).

### Measurement performance outcomes

Detailed results are shown in Tables 1 and 2, as well as in Supplementary Table S1.

**Table 1.** Differences Between Dexcom G5 Mobile Values and BGMS results as well as FreeStyle Libre Values and BGMS Results Based on Aggregated Data

	*DG5 (LOCF)*	*FLscan*
At times of therapeutic decisions,^a^ percentage of results exceeding		
15 mg/dL or 15%	24.3% (*n* = 694)	25.0% (*n* = 625)
CGM < BGM	60.4%	48.6%
CGM > BGM	39.6%	51.4%
20 mg/dL or 20%	14.3% (*n* = 410)	14.2% (*n* = 356)
CGM < BGM	59.8%	53.1%
CGM > BGM	40.2%	46.9%
30 mg/dL or 30%	5.2% (*n* = 149)	5.2% (*n* = 129)
CGM < BGM	60.4%	61.2%
CGM > BGM	39.6%	38.8%
50 mg/dL or 50%	0.7% (*n* = 20)	0.8% (*n* = 21)
CGM < BGM	40.0%	71.4%
CGM > BGM	60.0%	28.6%
Total number of results	2891	2503

^a^ Therapeutic decisions were defined as BG measurements at times of meal intake and/or insulin delivery.

DG5, Dexcom G5; FL, FreeStyle Libre; BGMS, blood glucose monitoring system; LOCF, last observed carried forward; BGM, blood glucose monitoring; BG, blood glucose; CGM, continuous glucose monitoring.

**Table 2.** Performance Parameters for the Dexcom G5 Mobile System and the FreeStyle Libre System When Compared Against BGMS Results Based on Aggregated Data

	*DG5 (INT)*	*DG5 (LOCF)*	*FLcont. (INT)*	*FLscan*
MAD/MARD (complete experiments) versus capillary BG^a^				
MAD (<100 mg/dL), in mg/dL	10.5 ± 10.7 (*n* = 2462)	11.6 ± 11.5 (*n* = 2485)	12.5 ± 11.0 (*n* = 2370)	13.2 ± 11.6 (*n* = 2318)
MARD (≥100 mg/dL)	10.3% ± 10.8% (*n* = 6991)	11.3% ± 11.5% (*n* = 7048)	10.5% ± 9.9% (*n* = 6486)	11.3% ± 10.6% (*n* = 6323)
MAD/MARD (in house phases), based on max. one value per hour, versus capillary BG^a,b^				
MAD (<100 mg/dL), in mg/dL	10.0 ± 11.1 (*n* = 947)	11.0 ± 11.9 (*n* = 952)	13.0 ± 11.1 (*n* = 932)	13.2 ± 11.7 (*n* = 929)
MARD (≥100 mg/dL)	9.4% ± 10.5% (*n* = 3516)	10.2% ± 11.1% (*n* = 3541)	10.4% ± 9.9% (*n* = 3324)	11.0% ± 10.4% (*n* = 3288)
MAD/MARD (dynamic phases) versus capillary BG^a^				
MAD (<100 mg/dL), in mg/dL	11.7 ± 12.2 (*n* = 822)	13.0 ± 13.2 (*n* = 835)	14.1 ± 11.5 (*n* = 775)	15.2 ± 12.4 (*n* = 771)
MARD (≥100 mg/dL)	12.4% ± 11.0% (*n* = 1752)	13.5% ± 12.1% (*n* = 1762)	12.3% ± 10.7% (*n* = 1622)	13.7% ± 11.9% (*n* = 1620)
MAD/MARD (dynamic phases) versus venous BG^a^				
MAD (<100 mg/dL), in mg/dL	13.1 ± 12.8 (*n* = 790)	14.4 ± 14.0 (*n* = 803)	14.9 ± 12.0 (*n* = 749)	15.9 ± 12.7 (*n* = 745)
MARD (≥100 mg/dL)	13.4% ± 11.6% (*n* = 1642)	14.7% ± 12.7% (*n* = 1652)	13.0% ± 11.4% (*n* = 1506)	14.7% ± 12.8% (*n* = 1508)
MAD/MARD (home use phases) versus capillary BG^a^				
MAD (<100 mg/dL), in mg/dL	10.0 ± 9.2 (*n* = 705)	10.9 ± 9.7 (*n* = 712)	10.4 ± 10.1 (*n* = 656)	11.5 ± 10.7 (*n* = 616)
MARD (≥100 mg/dL)	10.2% ± 10.1% (*n* = 1938)	11.1% ± 10.7% (*n* = 1958)	8.8% ± 8.4% (*n* = 1734)	9.6% ± 8.8% (*n* = 1622)
Consensus error grid (complete experiments)^a^				
Zone A	85.8% (*n* = 8114 of 9453)	83.5% (*n* = 7964 of 9533)	85.5% (*n* = 7575 of 8856)	83.6% (*n* = 7225 of 8641)
Zone B	13.8% (*n* = 1302 of 9453)	16.0% *n* = 1521 of 9533)	14.2% (*n* = 1261 of 8856)	16.0% (*n* = 1386 of 8641)
PAD/PARD (complete experiments)^c^				
PAD (<100 mg/dL), in mg/dL	8.3 ± 9.0 (*n* = 88172)	n.d.^d^	12.1 ± 12.8 (*n* = 85435)	n.d.^d^
PARD (≥100 mg/dL)	7.6% ± 8.3% (*n* = 289555)	n.d.^d^	9.6% ± 10.3% (*n* = 241358)	n.d.^d^

Because of differences between continuously stored and scanned FL data (FLcont and FLscan, respectively), separate analyses were performed.

^a^ Results given as mean ± standard deviation.

^b^ Comparison measurements were limited to up to one value per hour to not overrepresent the dynamic phases with one value every 15 min.

^c^ Analysis was performed on data with one value per minute obtained from linearly interpolating sensor readings.

^d^ Analysis was not performed.

INT, linearly interpolated data (interpolation of one value per minute); MAD, mean absolute difference; MARD, mean absolute relative difference; n.d., not done; PAD, precision absolute difference; PARD, precision absolute relative difference.

**FIG. 2.**
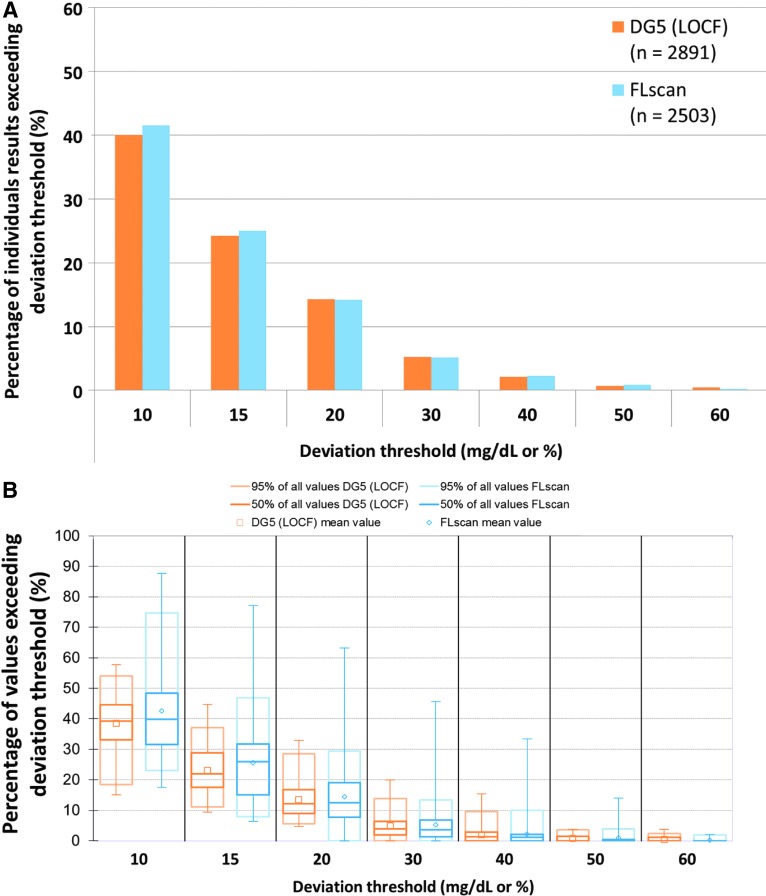
Deviations (differences and relative differences) between continuous tissue glucose monitoring readings and blood glucose monitoring system results at times of therapeutic decisions. **(A)** Aggregated data. **(B)** Box plots of application-site-specific percentages (*n* = 40 per plot); whiskers represent minimum and maximum values. DG5, Dexcom G5; FL, FreeStyle Libre; LOCF, last observed carried forward.

At times of therapeutic decisions, approximately one in four CGM readings (DG5: 24.3%, FL: 25.0% differed more than ±15 mg/dL or ±15% for BG <100 mg/dL or BG ≥100 mg/dL, respectively, from the corresponding BGMS results (Fig. 2A). Variability among the individual participants' application sites was higher in FL than in DG5 (Fig. 2B). For ∼1 in 20 readings (DG5: 5.2%, FL: 5.2%), the difference from the corresponding BGMS results exceeded ±30 mg/dL or ±30%.

**FIG. 3.**
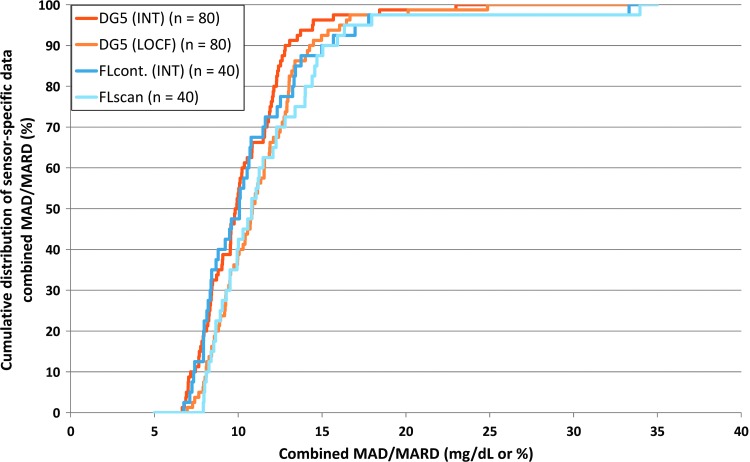
Cumulative distribution of combined MAD/MARD results of complete sensor experiments. FLcont, continuously stored FreeStyle Libre data; FLscan, scanned FreeStyle Libre data; INT, interpolated data; MAD, mean absolute difference; MARD, mean absolute relative difference.

MAD and MARD results for DG5 and for FL were similar. For DG5, results based on the LOCF approach were slightly higher (i.e., worse) than using interpolated data. For FL, results for FLscan were also slightly higher (i.e., worse) than for interpolated FLcont. Cumulative distribution of combined MAD/MARD results for individual sensor experiments is shown in Figure 3. During the first 24 h of CGM system usage, combined MAD/MARD results were higher than the other days (Supplementary Fig. S2), whereas BG concentration only slightly affected results (Supplementary Fig. S3). During dynamic phases with rapid glucose changes, MAD/MARD were higher for all systems than during the other phases, and there were qualitative differences in how well the systems coped with rising or falling glucose concentrations (Supplementary Fig. S4).

**FIG. 4.**
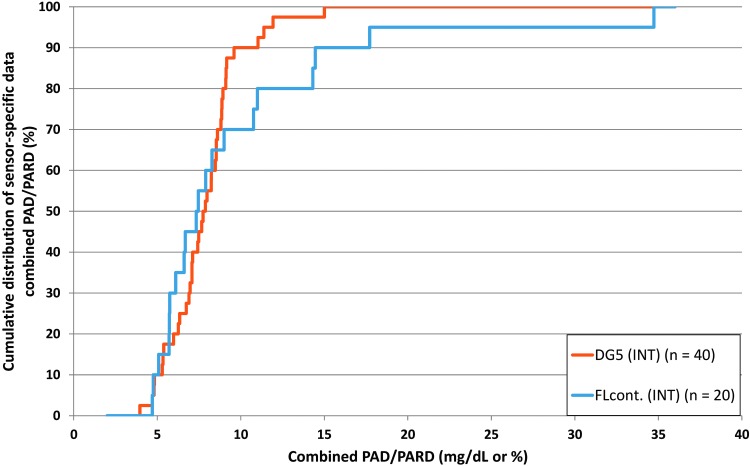
Cumulative distribution of combined PAD/PARD results of pairs of complete sensor experiments. PAD, precision absolute difference; PARD, precision absolute relative difference.

PAD and PARD results were lower (i.e., better) for DG5 than for FLcont. FLscan was not analyzed because the relatively sparse data did not allow for appropriate analysis of PAD/PARD. Cumulative distribution of combined PAD/PARD results is shown in Figure 4. MARD results and PARD results for the complete glucose concentration range are provided in Supplementary Table S2.

In the CEG analysis, 99.6%–99.8% of paired results were found within the clinically acceptable zones A and B (Supplementary Fig. S5).

### Technical and safety outcomes

For DG5, 12 DD were documented (10 regarding receivers, 1 regarding a transmitter, 1 regarding a sensor), 2 of which did lead to device replacements: One receiver displayed multiple error messages and technical support advised replacement. As the original transmitter could not be connected to the replaced receiver, it was also replaced on the next study site visit. This was categorized as a separate device replacement because technical support only advised replacement of the receiver.

For DG5, one sensor was removed prematurely because the transmitter stopped working (see above). This sensor was not replaced.

For FL, three DD were documented that led to two device replacements: For one sensor, the applicator did not work, and the sensor was replaced. Another sensor could not be scanned because of repeated sensor errors within the first hours of sensor usage. This sensor was also replaced. In the third case, which did not lead to replacement, the FL reader displayed that the sensor session had ended after 2.3 days of use.

In total, 7 of 40 FL sensors were removed prematurely: in one case, FL reader displayed that the sensor session had ended after 2.3 days of use (this was also categorized as DD); three sensors were inadvertently removed after 2.8, 4.9, and 12.0 days, respectively; and in three cases, the adhesive plaster was not able to attach the sensor to the skin for the entire duration of 14 days so that they fell off after 2.1, 10.0, and 10.3 days, respectively. These sensors were not replaced.

In total, 42 AE occurred in 17 participants during the study, 21 (50%) of which were unrelated to study procedures, for example, a cold or hematoma occurred from the participants' own insulin catheters or own CGMs worn before the start of the study. Of the other 21 (50%) AE, 13 (31% of all AE; 62% of procedure-related AE) qualified as ADE related to DG5 (6 ADE) or FL (7 ADE). Procedure-related AE without causal relationship to investigational devices were caused by the venous catheters (hematoma or pain at insertion sites). All of the 13 documented ADE are common in CGM usage, for example, hematoma and pain at insertion sites. None of the ADE was caused by DD of the investigational devices. No serious AE or serious ADE, and no other significant medical event occurred during the complete study. Details about ADE are shown in Supplementary Table S3.

## Discussion

DG5 and FL were compared in a study, in which 20 participants wore two sensors of each system for 14 days in a clinical setting and at home. Participants were instructed to base therapeutic decisions on BG measurements at all times. Regarding safety analysis of these systems, some AE had a causal relationship to the investigational devices, but these AE are common in CGM usage.

At times of therapeutic decisions, defined as carbohydrate intake and/or insulin delivery, approximately one in four readings of DG5 and FL differed from BG readings by at least ±15 mg/dL or ±15%, and ∼1 in 20 readings differed by at least ±30 mg/dL or ±30%. Thus, a considerable number of therapeutic decisions may have been made differently if they had been based on DG5 or FL readings instead of BG measurements. Although error grid analysis, which is a common approach for estimating clinical relevance of glucose monitoring systems, did find almost all measurement results to be clinically acceptable, an effect on the quality of diabetes therapy is possible. An in-silico study^19^ about the nonadjunctive use of CGM systems found that the effect on diabetes therapy is relevant in the range of MARD results observed in this study. Considering that users of these systems reportedly perform only few additional BG measurements (2.8 measurements per day for DG5,^20^ including two BG measurements for calibration, and 0.5 measurements per day for FL^21^), nonadjunctive use seems to be already practiced by some CGM users. Recent publications about the nonadjunctive use of these CGM systems in people with type 1 diabetes mellitus or type 2 diabetes mellitus reported no change in time spent in the glucose target range,^20–23^ suggesting that using CGM instead of BGM for therapeutic decisions might not affect the quality of diabetes therapy in the short term. Possible long-term effects, however, remain unclear.

Another finding was that DG5 showed slightly smaller deviations from BG readings ≥100 mg/dL than FL during in house phases, but FL showed slightly smaller deviations from BG readings ≥100 mg/dL during home use phases. This result could be influenced by several factors: During study site visits, participants were supervised by study staff to minimize possible BGMS and CGM system handling errors, including calibration errors. In addition, daily routines during study site visits were planned to minimize glucose rates of change at times of DG5 calibration. At home, however, participants may not have taken as much care in planning DG5 calibrations. Another possible influence is the difference in frequency of BGMS measurements between study site visits and home use phases.^24^

This study focused on the analytical point accuracy of the investigated CGM systems. Therefore, the study design did not allow for a universal comparison of the effect on the quality of therapeutic decisions. As mentioned before, CGM systems typically not only show a current glucose reading but also glucose trend information or a graph of previous glucose levels (glucose curve). If the user is sufficiently educated on the proper use of this information, a lower level of point accuracy of CGM systems compared with BGMS may still allow for adequate diabetes therapy.^25^

At the time this study was conducted, DG5 and FL have not yet been compared in a head-to-head setting. This comparison allowed a more detailed analysis than other performance assessments, because study design is a known influence factor in assessing CGM measurement performance by MARD.^26^ While DG5 and FL measurement performance results were comparable to what had been published previously for each of the systems separately,^1,2,27^ qualitative differences between DG5 and FL were found. Overall, DG5 showed slightly smaller deviations from BGMS results, for example, smaller percentage of differences exceeding specific thresholds or smaller MARD than FL, and interestingly, continuously stored FL data deviated to a slightly smaller extent from BGMS results than scanned FL data. This raises the question why scanned data differ from continuously stored data and how this may affect diabetes therapy.^28^

At times of (induced) rapid changes in glucose concentration, differences between DG5 results and BGMS results and between FL results and BGMS results increased in comparison to the overall results. This increase is likely affected by the time lag between BG changes and TG changes having a more pronounced effect in times of rapid glucose changes.

Judging by clinical relevance of measurement differences between DG5 or FL and BGMS results using the CEG, almost all differences were clinically acceptable (≥99.6% in zones A and B). However, 0.7% and 0.8% of DG5 and FL readings, respectively, exceeded a difference of ±50 mg/dL or 50% at times of therapeutic decisions, which could potentially have considerable effect on clinical action. It is noteworthy that there are multiple sets of requirements regarding analytical performance of BGMS, for example, the international standard ISO 15197^14^ or the FDA guidance document #1756 on BGMS for over-the-counter use.^29^ Even though the two investigated CGM systems are approved to replace BGM in many situations, performance requirements have not yet been established.

Diary records from this study indicate that the values displayed by DG5 are identical to the values stored in DG5 memory. Therefore, the LOCF approach might more adequately reflect the analytical quality of the CGM system as perceived by the user. As performance results for DG5 depended on whether linear interpolation or the LOCF approach was used, it should be discussed which approach is more appropriate in performance analyses.

Sensor-to-sensor precision was better (i.e., PAD/PARD values were smaller) in DG5 than in FL. It should be investigated to what degree sensor-to-sensor variability affects the quality of diabetes therapy, because users may not know if they happen to use a “good” or a “bad” sensor.

## Conclusions

In summary, approximately one in four values as displayed by DG5 and FL deviated from the corresponding BGM readings by at least 15 mg/dL or 15%, and 1 in 20 values deviated by at least 30 mg/dL or 30%. DG5 and FL exhibited mostly comparable deviations from BGMS results in this study. This is in contrast to CEG analysis, according to which only a small percentage of results could have had considerable effect on clinical action. DG5 results deviated slightly less from BGMS results when looking at the complete study and at the well-defined setting of the study site visits, whereas FL results deviated to a slightly smaller extent during home use phases.

## Supplementary Material

Supplemental data

Supplemental data

Supplemental data

Supplemental data

Supplemental data

Supplemental data

Supplemental data

Supplemental data

Supplemental data
