# Supplementary material for: Measurement Performance of Two Continuous Tissue Glucose Monitoring Systems Intended for Replacement of Blood Glucose Monitoring
Source: Diabetes Technol Ther. 2018 Aug 1;20(8):541–9. doi: 10.1089/dia.2018.0105 (PMC6080122; doi:10.1089/dia.2018.0105)
Supplement: Supplemental data [file Supp_Fig1.pdf]

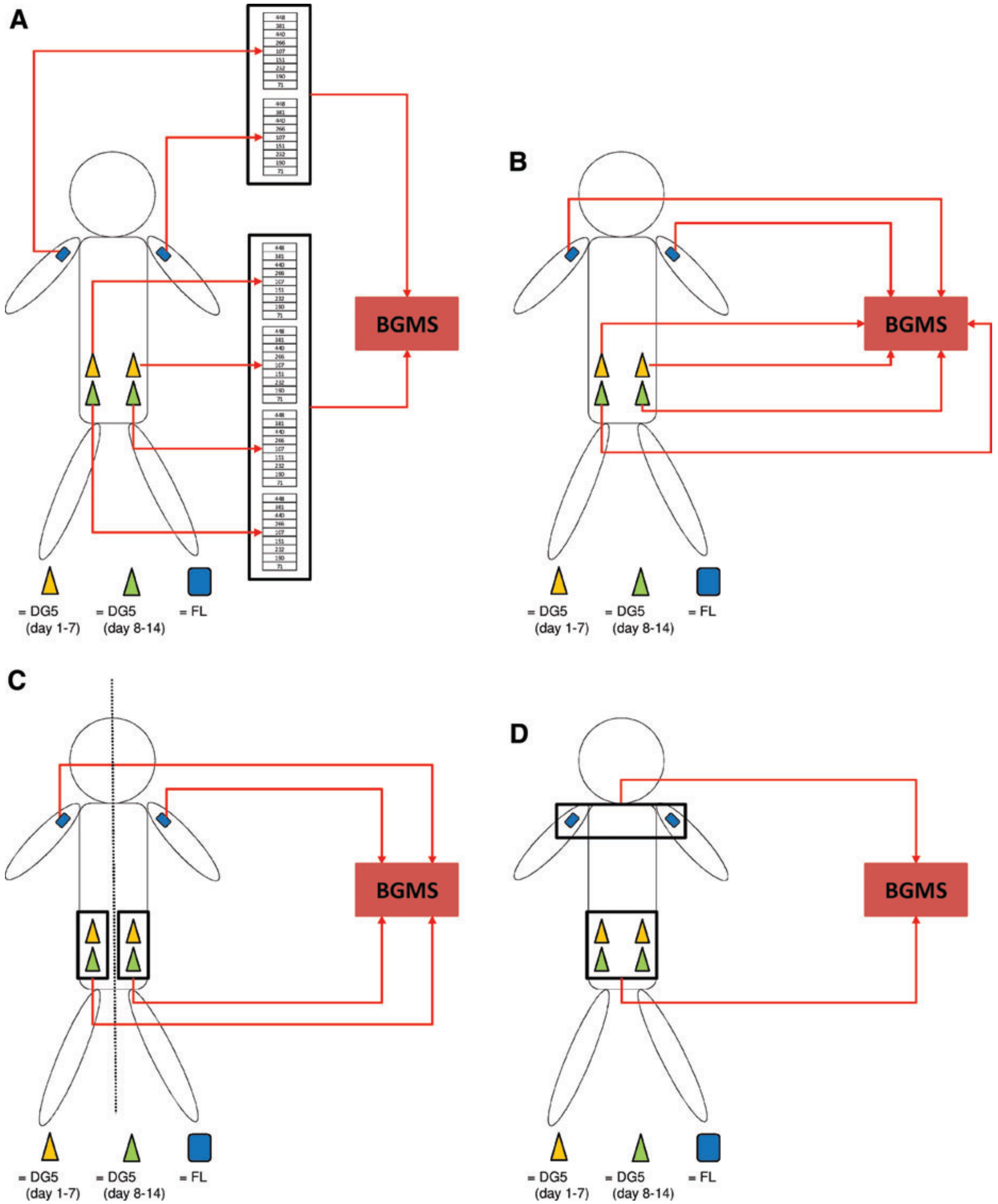

SUPPLEMENTARY FIG. S1. Evaluation units. **(A)** Aggregated data. **(B)** Grouped by sensor. **(C)** Grouped by each individual participant's application sites. **(D)** Grouped by participant. BGMS, blood glucose monitoring system; DG5, Dexcom G5; FL, FreeStyle Libre.
