# Supplementary material for: Measurement Performance of Two Continuous Tissue Glucose Monitoring Systems Intended for Replacement of Blood Glucose Monitoring
Source: Diabetes Technol Ther. 2018 Aug 1;20(8):541–9. doi: 10.1089/dia.2018.0105 (PMC6080122; doi:10.1089/dia.2018.0105)
Supplement: Supplemental data [file Supp_Data.pdf]

Supplementary Data

Inclusion and exclusion criteria

Inclusion criteria

- Patients with type 1 diabetes on multiple daily injections or continuous subcutaneous insulin infusion according to American Diabetes Association/World Health Organization-definition
- Age ≥18 years
- Age >45 years: profound examination with cardiovascular risk assessment
- Signed and dated Informed Consent Form

Exclusion criteria

- Age >65 years
- Age >45 years: patients with at least one additional risk factor for cardiovascular diseases, for example, smoking, treated arterial hypertension, or systolic blood pressure >140 mmHg or diastolic blood pressure >90 mmHg), hyperlipidemia (elevated total cholesterol, elevated low-density lipoprotein cholesterol), HbA1c >8.5%
- Severe acute or chronic illness besides diabetes mellitus or history of any illness that, in the opinion of the investigator, might confound the results of the study or pose additional risk in applying the medical device to the patient
- Pregnancy or lactation period
- Significantly impaired awareness of hypoglycemia
- Severe skin abnormalities or skin diseases, for example, psoriasis vulgaris, at the potential sensor insertion sites
- Mental incapacity or language barriers precluding adequate compliance with the study procedures
- Legal incompetence or limited legal competence
- Dependency from the sponsor or the clinical investigator (e.g., employees of the sponsor or the study site or their families)
