# Supplementary material for: Measurement Performance of Two Continuous Tissue Glucose Monitoring Systems Intended for Replacement of Blood Glucose Monitoring
Source: Diabetes Technol Ther. 2018 Aug 1;20(8):541–9. doi: 10.1089/dia.2018.0105 (PMC6080122; doi:10.1089/dia.2018.0105)
Supplement: Supplemental data [file Supp_Table1.pdf]

Supplementary Table S1. Measurement Accuracy for Complete Sensor Experiments of Dexcom G5 and FreeStyle Libre

Results within accuracy limits	*DG5 (INT) (%)*	*DG5 (LOCF) (%)*	*FLcont (INT) (%)*	*FLscan (%)*
30 mg/dL or 30%	95.1	93.8	94.5	93.3
20 mg/dL or 20%	86.4	84.1	85.5	83.1
15 mg/dL or 15%	77.0	73.9	75.4	72.4
10 mg/dL or 10%	61.3	58.1	58.1	55.5
N	9453	9533	8856	8641

For DG5, linearly interpolated data (INT) and "last observed carried forward" (LOCF) data are shown. For FL, linearly interpolated continuously stored data [FLcont (INT)] and scanned data (FLscan) are shown. The study blood glucose monitoring system was used as comparison method. Differences are calculated at blood glucose results <100 mg/dL, relative difference are calculated at blood glucose results ≥100 mg/dL.

DG5, Dexcom G5; FL, FreeStyle Libre.
