# Supplementary material for: Measurement Performance of Two Continuous Tissue Glucose Monitoring Systems Intended for Replacement of Blood Glucose Monitoring
Source: Diabetes Technol Ther. 2018 Aug 1;20(8):541–9. doi: 10.1089/dia.2018.0105 (PMC6080122; doi:10.1089/dia.2018.0105)
Supplement: Supplemental data [file Supp_Fig2.pdf]

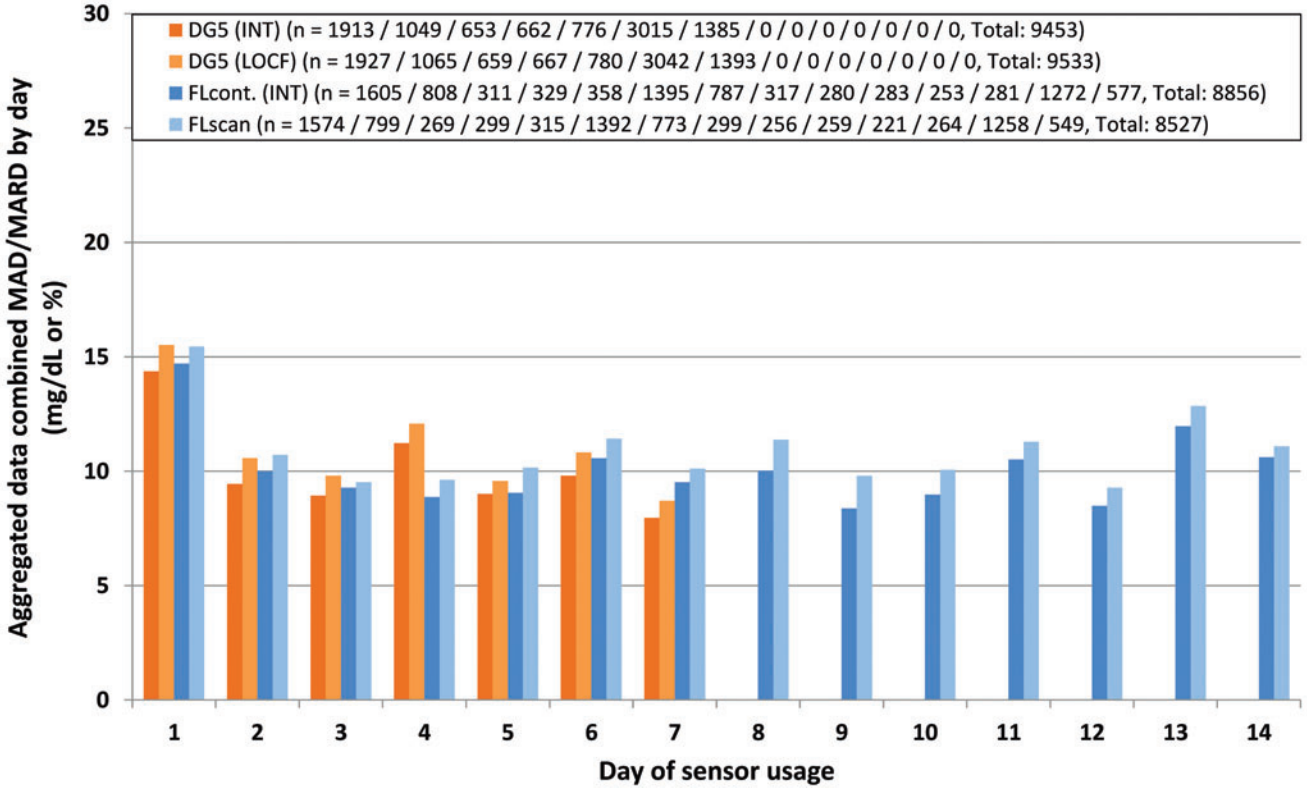

SUPPLEMENTARY FIG. S2. Combined MAD/MARD results for complete experiments by day of sensor usage for the DG5 (Dexcom, Inc., San Diego, CA) system and the FL (Abbott Diabetes Care, Alameda, CA) system. For DG5, linearly INT and LOCF data are shown. For FL, linearly interpolated continuously stored data [FLcont (INT)] and scanned data (FLscan) are shown. MAD for blood glucose concentrations <100 mg/dL, MARD for blood glucose concentrations ≥100 mg/dL. The study blood glucose monitoring system was used as comparison method. INT, interpolated data; LOCF, last observed carried forward; MAD, mean absolute difference; MARD, mean absolute relative difference.
