# Supplementary material for: Measurement Performance of Two Continuous Tissue Glucose Monitoring Systems Intended for Replacement of Blood Glucose Monitoring
Source: Diabetes Technol Ther. 2018 Aug 1;20(8):541–9. doi: 10.1089/dia.2018.0105 (PMC6080122; doi:10.1089/dia.2018.0105)
Supplement: Supplemental data [file Supp_Fig3.pdf]

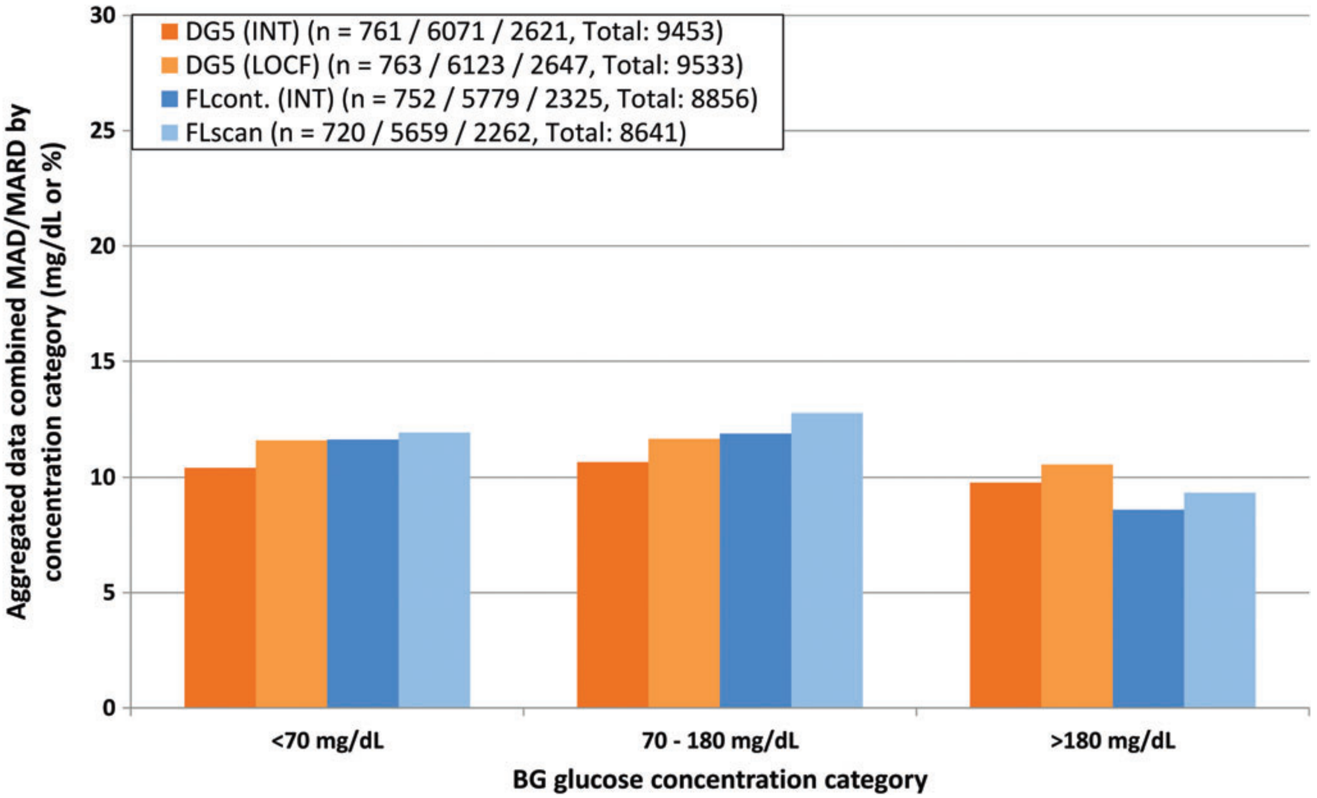

SUPPLEMENTARY FIG. S3. Combined MAD/MARD results for complete experiments by concentration category for DG5 and FL. For DG5, linearly INT and LOCF data are shown. For FL, linearly interpolated continuously stored data [FLcont (INT)] and scanned data (FLscan) are shown. MAD for blood glucose concentrations <100 mg/dL, MARD for blood glucose concentrations ≥100 mg/dL. The study blood glucose monitoring system was used as comparison method.
