# Supplementary material for: Measurement Performance of Two Continuous Tissue Glucose Monitoring Systems Intended for Replacement of Blood Glucose Monitoring
Source: Diabetes Technol Ther. 2018 Aug 1;20(8):541–9. doi: 10.1089/dia.2018.0105 (PMC6080122; doi:10.1089/dia.2018.0105)
Supplement: Supplemental data [file Supp_Table2.pdf]

Supplementary Table S2. Mean Absolute Relative Deviation and Precision Absolute Relative Deviation Results for Dexcom G5 and FreeStyle Libre for the Complete Glucose Concentration Range

	DG5 (INT)	*DG5 (LOCF)*	*FLcont. (INT)*	*FLscan*
MARD (complete experiments) versus capillary BG[a]	11.4% ± 12.2% (n = 9453)	12.4% ± 13.1% (n = 9533)	12.2% ± 11.9% (n = 8856)	13.0% ± 12.5% (n = 8641)
MARD (in house phases), based on max. one value per hour, versus capillary BG[a,b]	10.2% ± 11.8% (n = 4463)	11.1% ± 12.6% (n = 4493)	11.8% ± 11.3% (n = 4256)	12.3% ± 11.7% (n = 4217)
MARD (dynamic phases) versus capillary BG[a]	13.4% ± 13.5% (n = 2574)	14.7% ± 14.6% (n = 2597)	14.4% ± 12.5% (n = 2397)	15.7% ± 13.4% (n = 2391)
MARD (dynamic phases) versus venous BG[a]	14.8% ± 14.6% (n = 2432)	16.2% ± 16.1% (n = 2455)	15.3% ± 13.6% (n = 2255)	16.8% ± 14.7% (n = 2253)
MARD (home use phases) versus capillary BG[a]	11.2% ± 11.2% (n = 2643)	12.3% ± 12.0% (n = 2670)	10.5% ± 11.6% (n = 2390)	11.4% ± 12.1% (n = 2238)
PARD (complete experiments)[c]	8.3% ± 9.3% (n = 377,727)	n.d.[d]	11.2 ± 12.5% (n = 326,793)	n.d.[d]

For DG5, linearly interpolated data (INT) and ‘‘last observed carried forward’’ (LOCF) data are shown. For FL, linearly interpolated continuously stored data [FLcont (INT)] and scanned data (FLscan) are shown. The study blood glucose monitoring system was used as comparison method.

[a]Results given as mean ± standard deviation.

[b]Comparison measurements were limited to up to one value per hour to not overrepresent the dynamic phases with one value every 15 minutes.

[c]Analysis was performed on data with one value per minute obtained from linearly interpolating sensor readings.

[d]Analysis was not performed.

BG, blood glucose; MAD, mean absolute difference; MARD, mean absolute relative difference; PARD, precision absolute relative difference.
