# Supplementary material for: Measurement Performance of Two Continuous Tissue Glucose Monitoring Systems Intended for Replacement of Blood Glucose Monitoring
Source: Diabetes Technol Ther. 2018 Aug 1;20(8):541–9. doi: 10.1089/dia.2018.0105 (PMC6080122; doi:10.1089/dia.2018.0105)
Supplement: Supplemental data [file Supp_Fig5.pdf]

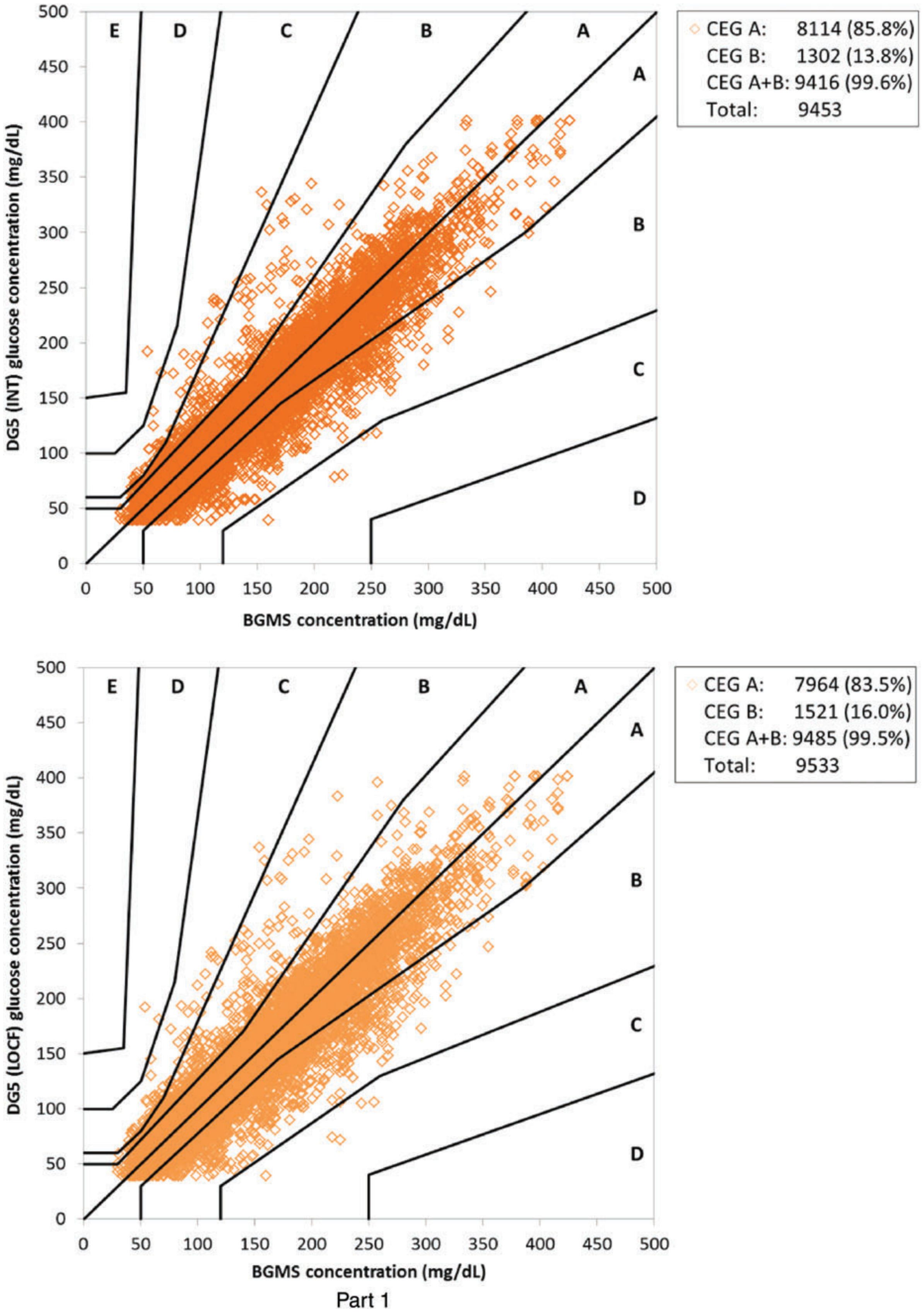

SUPPLEMENTARY FIG. S5. CEG analysis for DG5 and FL. For DG5, linearly INT and LOCF data are shown. For FL, linearly interpolated continuously stored data [FLcont (INT)] and scanned data (FLscan) are shown. Measuring range: 40–400 mg/dL for DG5, 40–500 mg/dL for FL. The study blood glucose monitoring system was used as comparison method. Due to rounding, the sum of CEG A percentage and CEG B percentage may differ from CEG A + B percentage. CEG, consensus error grid.

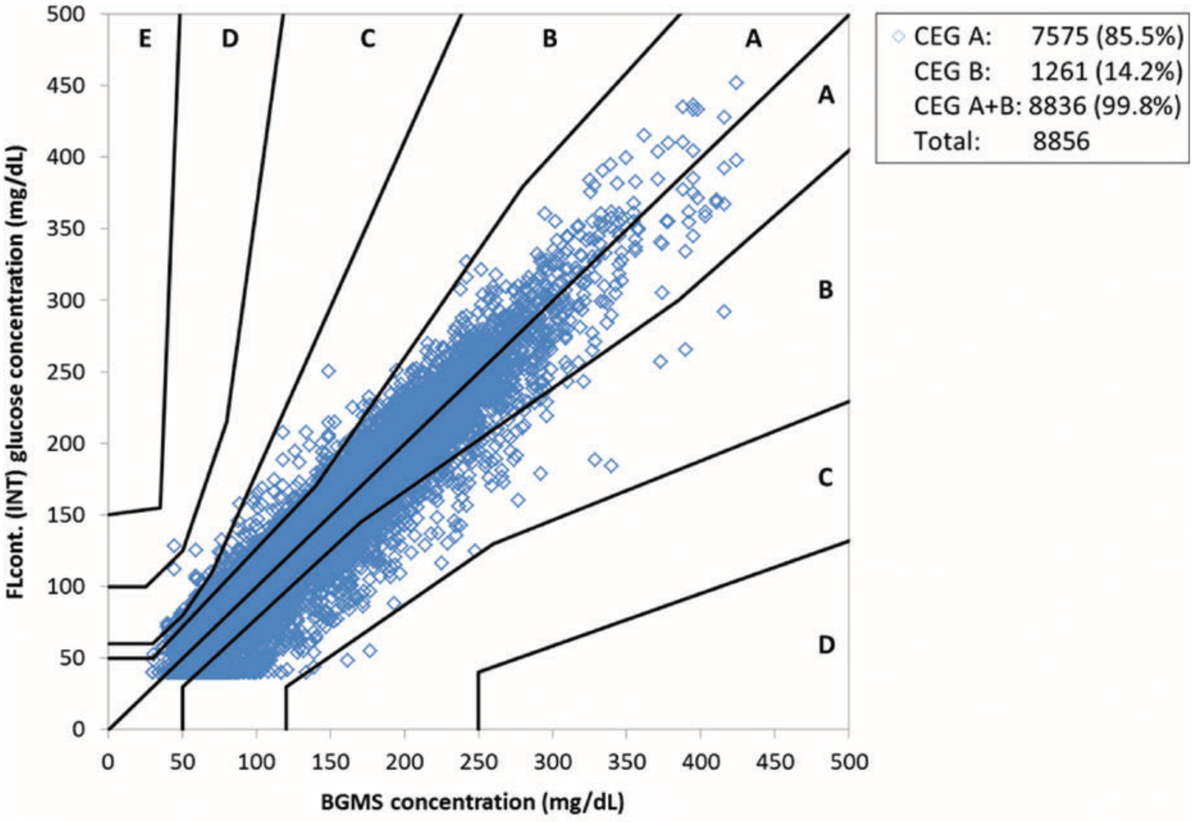

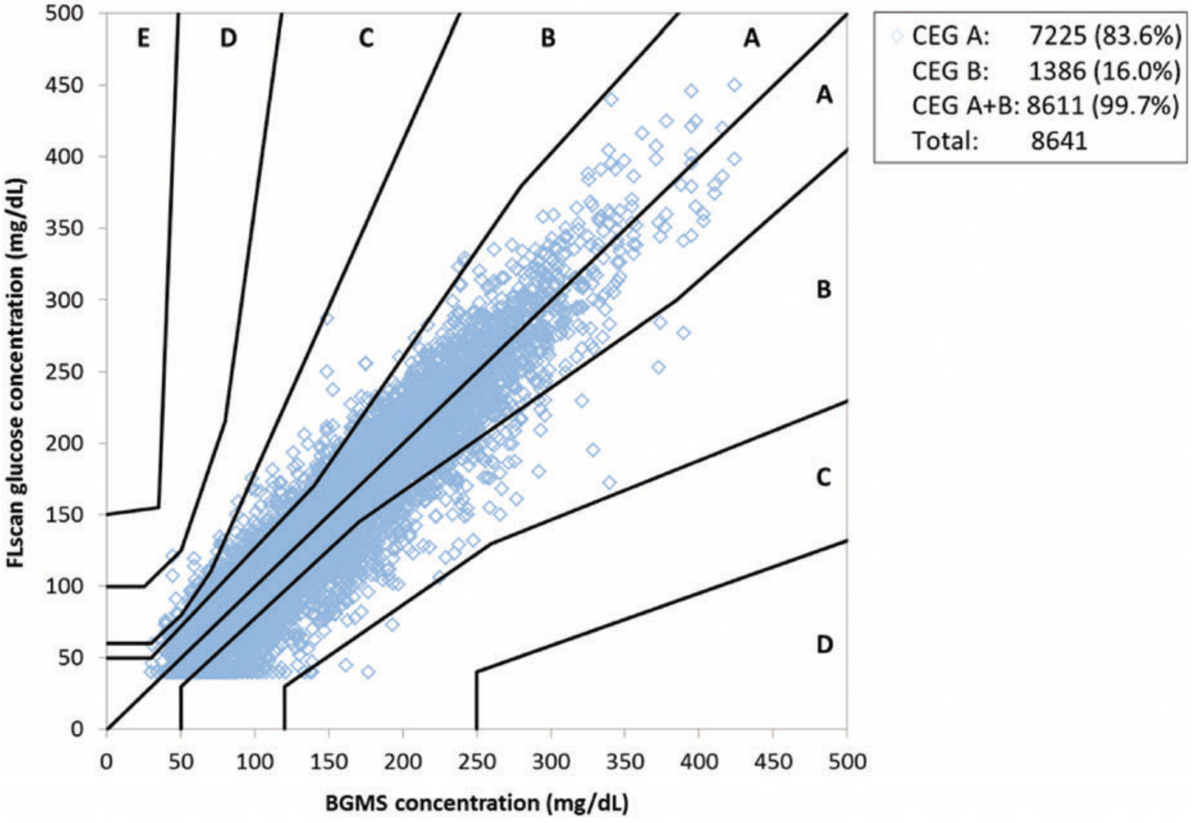

Part 2

SUPPLEMENTARY FIG. S5. (Continued).
