# Supplementary material for: Measurement Performance of Two Continuous Tissue Glucose Monitoring Systems Intended for Replacement of Blood Glucose Monitoring
Source: Diabetes Technol Ther. 2018 Aug 1;20(8):541–9. doi: 10.1089/dia.2018.0105 (PMC6080122; doi:10.1089/dia.2018.0105)
Supplement: Supplemental data [file Supp_Table3.pdf]

Supplementary Table S3. List of Adverse Device Effects

Participant #	*DG5*	*FL*	*Description*
01		x	Skin reaction (redness and pressure mark) with light maceration
01		x	Skin reaction (redness and pressure mark)
02	x		Itching
03		x	Skin reaction (redness and pressure mark)
05	x		Pain, sensor insertion site
05	x		Pain, sensor insertion site
05		x	Pain, sensor insertion site
07	x		Hematoma, sensor insertion site
09	x		Hematoma, sensor insertion site
11		x	Hematoma, sensor insertion site
13		x	Pain, sensor insertion site
13		x	Pain, sensor insertion site
19	x		Hematoma, sensor insertion site
Sum	6	7	

All adverse device effects were classified as mild and nonserious.
